# Disentangling molecular relationships with a causal inference test

**DOI:** 10.1186/1471-2156-10-23

**Published:** 2009-05-27

**Authors:** Joshua Millstein, Bin Zhang, Jun Zhu, Eric E Schadt

**Affiliations:** 1 Genetics Dept., Rosetta Inpharmatics, LLC, wholly owned subsidiary of Merck & Co., Inc., 401 Terry Avenue North, Seattle Washington, 98109, USA

## Abstract

**Background:**

There has been intense effort over the past couple of decades to identify loci underlying quantitative traits as a key step in the process of elucidating the etiology of complex diseases. Recently there has been some effort to coalesce non-biased high-throughput data, e.g. high density genotyping and genome wide RNA expression, to drive understanding of the molecular basis of disease. However, a stumbling block has been the difficult question of how to leverage this information to identify molecular mechanisms that explain quantitative trait loci (QTL). We have developed a formal statistical hypothesis test, resulting in a p-value, to quantify uncertainty in a causal inference pertaining to a measured factor, e.g. a molecular species, which potentially mediates a known causal association between a locus and a quantitative trait.

**Results:**

We treat the causal inference as a 'chain' of mathematical conditions that must be satisfied to conclude that the potential mediator is causal for the trait, where the inference is only as good as the weakest link in the chain. P-values are computed for the component conditions, which include tests of linkage and conditional independence. The Intersection-Union Test, in which a series of statistical tests are combined to form an omnibus test, is then employed to generate the overall test result. Using computer simulated mouse crosses, we show that type I error is low under a variety of conditions that include hidden variables and reactive pathways. We show that power under a simple causal model is comparable to other model selection techniques as well as Bayesian network reconstruction methods. We further show empirically that this method compares favorably to Bayesian network reconstruction methods for reconstructing transcriptional regulatory networks in yeast, recovering 7 out of 8 experimentally validated regulators.

**Conclusion:**

Here we propose a novel statistical framework in which existing notions of causal mediation are formalized into a hypothesis test, thus providing a standard quantitative measure of uncertainty in the form of a p-value. The method is theoretically and computationally accessible and with the provided software may prove a useful tool in disentangling molecular relationships.

## Background

It has become increasingly appreciated in recent years that empirical evidence of causal links between genotype and multiple quantitative traits such as transcript abundances and clinical phenotypes can provide information on causal relationships between those quantitative traits [1-10]. The conceptual foundation of the inferred causal relationships is built on the idea that random segregation of chromosomes during gametogenesis insulates against confounding in a manner analogous to treatment randomization in a clinical trial [11,12]. Markov properties and conditional correlation have also been utilized by some investigators to disentangle the causal pathway, already simplified by the establishment of genotype as the origin of propagation [1,7,9,10,13-15]. Generally, causality is inferred after a series of mathematical conditions are met, but quantifying uncertainty in the causal call has been challenging.

Causal effect estimates often considered in 'Mendelian randomization' approaches [11], can be confounded by pleiotropic effects and reverse causation [12], thus, these approaches are not generally considered for problems such as reconstructing transcript regulatory pathways, in which pleiotropy is common and there may be little a priori information on the structure of the causal relationship between traits.

It's hard to overstate the importance of this problem as emerging technology and increased understanding of molecular biology allows cost-effective high-throughput quantification of multiple classes of molecular traits such as gene expression, CNV, miRNA, metabolite levels, splice variants, methylation, and protein expression. Designing and orchestrating classical studies to learn about causal pathways by perturbing individual factors would undoubtedly prove to be glacially slow given the extremely high dimensional nature of the problem.

Here we propose a statistical test to infer causal status for a potential mediator between a locus and a quantitative trait based on a set of mathematical conditions, thus providing a causal call and a quantitative measure of uncertainty in the causal inference in the form of a formal p-value. By 'causal', we mean that variance in the mediator determines some proportion of variance in the trait, even if that proportion is small. While the proposed approach does rely on some distributional and linear assumptions, generalizations to nonparametric and nonlinear models are straightforward.

## Results

### Conditions for causality

A genotype marker at a specific locus is denoted by L, transcript abundance for a specific transcript by G, and a measured clinical trait by T. Schadt [7] described a model selection approach based on conditional correlation, in which causality can be inferred if four conditions are met; 1) L and G are associated, 2) L and T are associated, 3) L is associated with G|T, and 4) L is independent of T|G. In practice, the first two conditions are often implicit, because statistical significance of a QTL is usually assessed at the peak marker, whereas the actual marker chosen to conduct a test of causality may be less statistically significantly associated with either or both traits. Here L is assumed to be sufficiently randomized by random segregation (and fixed due to the properties of DNA), thus L is analogous to a randomized treatment in a clinical trial in that association with L implies causation.

### Causal inference test (CIT)

Chen [1] presented theoretical evidence in the form of a 'Causality Equivalence Theorem' that causality is implied if three of the former conditions are satisfied under the assumption that L is randomized, specifically, 1) L and G are associated, 2) L and T are associated, and 3) L is independent of T|G. Although not included among their minimum conditions required to establish a causal relationship, Chen [1] presented evidence that the association of G with T|L is a necessary but not sufficient condition. It should be noted that a reactive model is consistent with the condition that L is independent of G|T, whereas the causal model is consistent with the condition that L is associated with G|T. This condition is not an explicit part of the Causality Equivalence Theorem, but the reasoning behind the proof is equally valid if we substitute condition 1, that L is associated with G, with the more stringent condition that L is associated with G|T. This condition is explicitly tested by Schadt [7] in their condition 3 and can provide additional information useful in distinguishing the causal from the reactive model. We took a conservative approach and included all non-redundant mathematical conditions from the previous discussion that are necessary and sufficient as a group to imply a causal relationship as our final suite of conditions that serve as a working mathematical definition of causality. To summarize, the conditions are 1) L and T are associated, 2) L is associated with G|T, 3) G is associated with T|L, and 4) L is independent of T|G.

### Component statistical tests

Each mathematical condition is assessed with a corresponding statistical test. Biallelic markers were considered here and fully modeled using two indicator covariates, L_1 _and L_2_, for one and two variant alleles, respectively, in a co-dominant coding. The four conditions are tested in the parameters of the following three linear regression models,(1)(2)(3)

where the *ε*_*ji *_represent independent random noise with variance . It should be noted that dependencies are likely to exist between certain pairs of covariates in the preceding three models. For instance, if the underlying relationship between variables is causal, reactive, or independent, dependencies will exist between T and L in model 2 and G and L in model 3. It precisely these dependencies we hope to learn about by conducting conditional tests of parameters. In terms of the preceding three models, the four component tests are:

For conditions 1 through 3, we use standard F tests for linear model coefficients, where the null hypotheses correspond to each set of parameters equaling zero. The F tests are conditional on the remaining terms in the model, that is, partial F tests are conducted for tests of parameters in equations (2) and (3). For the forth condition, the alternative hypothesis is {*β*_7_, *β*_8_} = 0 (conditional on the *β*_6 _term), which is an equivalence testing problem, that is, a non-significant test of the null that {*β*_7_, *β*_8_} = 0 does not correspond to statistically significant equivalence. Generally, in setting up an equivalence test, it is necessary to specify critical bounds that define a region in which the parameter is sufficiently close to the target value as to make any difference practically irrelevant. Chen et al. [1] in estimating the posterior probability of the condition that L is independent of T|G, defined this region as the parameter space that is closer to zero than we would expect under the 'independence' model. Here the 'independence' model is defined by three conditions, 1) L is causal for G, 2) L is causal for T, and 3) T is independent of G|L. Similarly, here we estimate the null distribution of the test statistic conditional on the observed T and the independence model, and test whether the observed statistic is significantly smaller than we would expect under the null.

**Figure 1:**
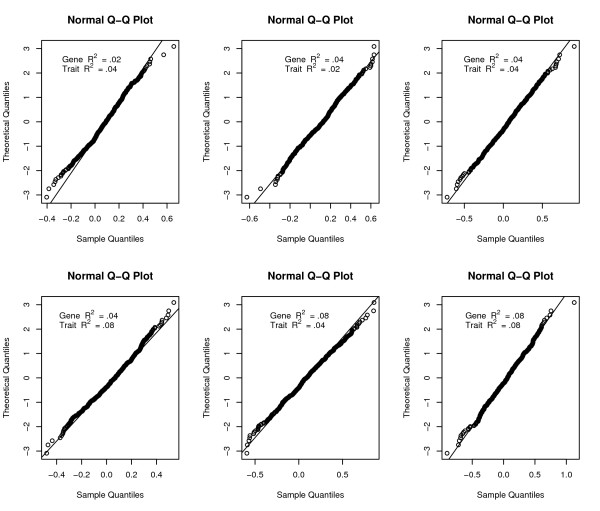
Estimated distributions of the test statistic, Z*, under the null for the equivalence test of conditional independence between the locus and the trait. An additive effect for a single biallelic locus under a simple independence model was simulated for both the gene and the trait under normally distributed errors. Minor allele frequency = .2. Sample size = 1000. B = 500.

**Figure 2:**
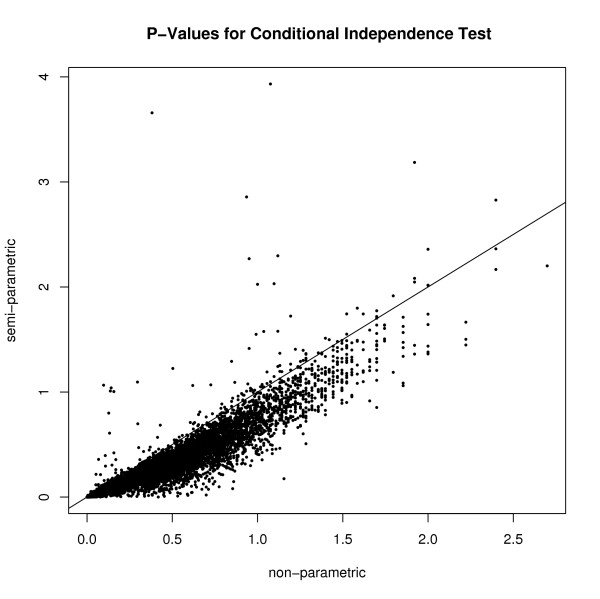
**Negative log 10 p-values for the semi-parametric and non-parametric versions of the CIT applied to 10,000 replicate data sets simulated under the independence model**. For each replicate of 100 observations the genetic variance for the gene and the trait were each randomly sampled from a uniform distribution ranging from 8 to 32 percent. The gene and trait were normally distributed and a biallelic locus was simulated with allele frequency of .5.

More specifically, the null distribution is estimated using a bootstrap type approach that consists of the following steps: 1) A random variable G* is simulated according to the marginal effect of L on G, thereby breaking any residual dependence between T and G|L. First, the marginal effect of L on G is estimated from the regression equation *G *= *α *+ *β*_1_*L*_1 _+ *β*_2_*L*_2 _+ . Then the residuals are randomly permuted and used with the estimated parameters to obtain G*. 2) The following two nested models

are fitted and a single realization of the test statistic, F*, is computed under the null distribution. 3) Steps 1 and 2 are repeated B times to create an empirical distribution of F* under the independence model. With B sufficiently large, the observed test statistic *F *can be tested directly against the empirical distribution of F* using a non-parametric permutation-based testing approach. Alternatively, if computation time is limiting, parametric properties of *F *in combination with a small B sample of F* can be used in a semi-parametric approach to approximate the null distribution. First, a method-of-moments approach yields an estimator for the non-centrality parameter of the F distribution under the null as,

where *ν*_1 _and *ν*_2 _denote the degrees of freedom. However, conditional on the observed T, the distribution has less spread than the unconditional distribution, so a standard *F *test here would result in an overly conservative estimate. To mitigate this problem we can transform to a normal distribution,

where , and we can estimate the variance, *σ*^2^, from the empirical distribution of Z*. Thus,

That is, the transformed observed statistic *Z *is tested against a  distribution. Figure 1 demonstrates that under the independence model, the normal distribution is a decent approximation for Z*. Figure 2 demonstrates that the semi-parametric approach is consistently more conservative than the non-parametric approach under the independence model.

### Omnibus test

The CIT can be thought of as testing the strength of a chain of mathematical conditions that as a set are consistent with causal mediation. Thus, the strength of the chain is only as strong as the weakest link, and correspondingly, the rejection region of the omnibus test is the intersection of rejection regions of the component tests. Cassela and Berger [16] showed that for a series of tests, each of size *α*_*γ *_and with rejection region *R*_*γ*_, then the 'intersection-union' test with rejection region ∩*R*_*γ *_is a level sup(*α*_*γ*_) test. Thus, the p-value for the CIT corresponds to the p-value for an intersection-union test, which is the supremum of the four p-values for the component tests.

**Figure 3:**
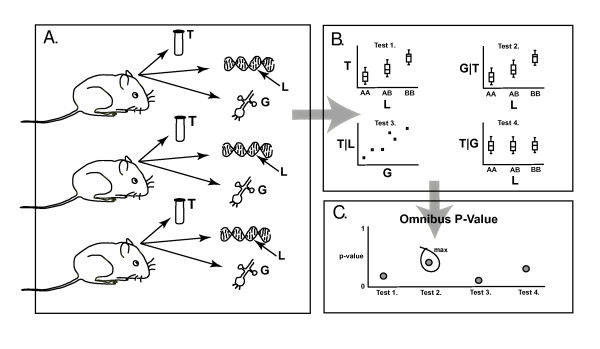
**Schematic diagram of the CIT**. A) Study subjects are sampled for i) a trait of interest, T (for example, cholesterol or fat mass), ii) a potential mediating factor, G (for example, an mRNA or protein concentration), and iii) genotype at a polymorphic locus, L, that is thought to affect both G and T. B) The four component tests of the CIT are conducted yielding four corresponding p-values. (Plots are shown as a conceptual device, see text for details of the actual tests.) Associations in 1 through 3 but not 4 are consistent with causal mediation. C) The largest of the four p-values becomes the omnibus p-value, the final result of the CIT.

A typical experiment where one might attempt to quantify causal associations between genes and clinical traits is an F2 cross setting where clinical traits, high density genotyping and genome wide RNA expression are collected. Our fundamental goal is to link all of this information in a meaningful way such that we can predict the genes that drive the traits of interest. More specifically, our goal here was to construct a robust statistical test to assess the hypothesis that a potential mediator between an initial randomized variable and an outcome variable is causal for that outcome. To achieve this objective we first identified a suite of mathematical conditions that could serve as a working mathematical definition of causality, could be formally statistically tested, and could support currently held views on minimum requirements necessary for causal inference. Second, we choose a method to compute a p-value for each condition. And third, we used the Intersection-Union Test framework [16] to compute an omnibus p-value for the suite of conditions that would function as a causal inference test (CIT) (Figure 3).

### CIT algorithm testing

Performance of the CIT was evaluated against three model selection approaches using simulated data from an F2 mouse cross measured on a genome wide high density panel of 2310 SNPs. The genotype data were simulated using RQTL software [17]. For each simulated replicate dataset, a segregating F2 population of 300 individuals was generated such that each of the 19 autosomes included a clinical trait QTL (cQTL) that was also a gene expression QTL (eQTL). Errors for both the clinical and expression traits were sampled from a standard normal distribution, and trait-trait associations were generated according to linear models. Single point linkage analysis was conducted for both the clinical trait and the gene expression trait. For each trait, the peak marker was identified by the maximum lod score across the chromosome. Causality tests were conducted at the clinical trait peak marker for all analyses reported here (tests for the 'reactive' model were conducted at the gene peak marker) unless stated otherwise. We would have preferred to compare the CIT to other hypothesis testing procedures for causal mediators. However, we were unaware of the existence of any other such methods; hence we have created a model selection approach from the CIT in order to compare it to other existing model selection based causal inference methods. The p-value was computed for both the causal and reactive models. If the causal p-value was < .05 and the reactive was > .05 then the call was causal. If both p-values were > .05 then the call was independent, and if both were < .05, then the call was 'no call.' Here 'independent' denotes the independent model as well as other non-causal and non-reactive models such as no genotype-phenotype association.

#### Model selection approaches for comparison

##### CC

The first model selection method, denoted by CC, is based on the conditional correlation approach [7] described in Methods. If the p-value for the partial F test for {*β*_4_, *β*_5_}, equation 2, is less than .05 and the p-value for the partial F for {*β*_7_, *β*_8_}, equation 3, is greater than .05, then the causal model is selected; if the converse is true, the reactive model is selected; if both p-values are below .05, the independence model is selected; and if both p-values are above .05 then no call is made.

##### AIC

The second approach, which depends on AIC values computed for regression models corresponding to the independence, reactive, and causal models, implemented by Chen et al. [1] is essentially a simplified version of the likelihood model selection approach described by Schadt et al. [7]. The AIC is computed for four regression models, m1) the trait regressed on the locus, equation 1 above, m2) the gene regressed on the locus, m3) the trait regressed on the gene, and m4) the gene regressed on the trait. The independence model corresponds to m1 and m2, the reactive model to m2 and m4, and the causal model to m1 and m3. The sum of the corresponding AIC values for each pair is computed, and the causation relationship with the lowest sum is selected.

##### BNC

The third method, denoted by BNC, employs the Bayesian Score, which is estimated for three networks, each including exactly two arcs, corresponding to the independent, the reactive, and the causal models (Figures 3A, B, and 3C). The independence network includes two arcs directed from the locus to the gene and the trait; the reactive model includes an arc directed from the locus to the trait and one from the trait to the gene; and the causal model includes an arc from the locus to the gene and one from the gene to the trait. The Bayesian score was computed using the DEAL software package [18].

**Figure 4:**
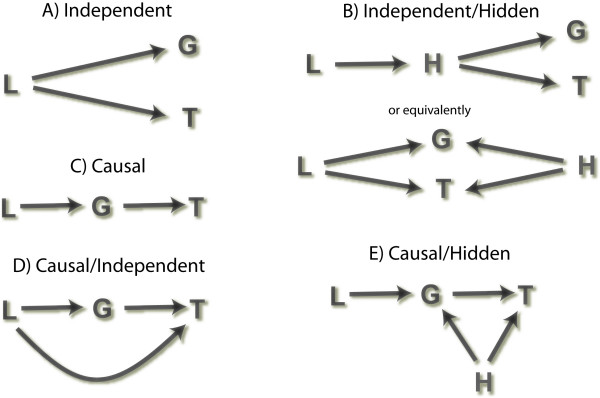
**Four causal inference strategies, CC, CIT, BNC, and AIC were applied to simulated data under five distinct causal models, A-E, shown above**. Here a genotype marker at a specific locus is denoted by L, a gene corresponding to measured transcript abundance is denoted by G, and a measured clinical trait is denoted by T. H denotes an unmeasured molecular trait.

**Figure 5:**
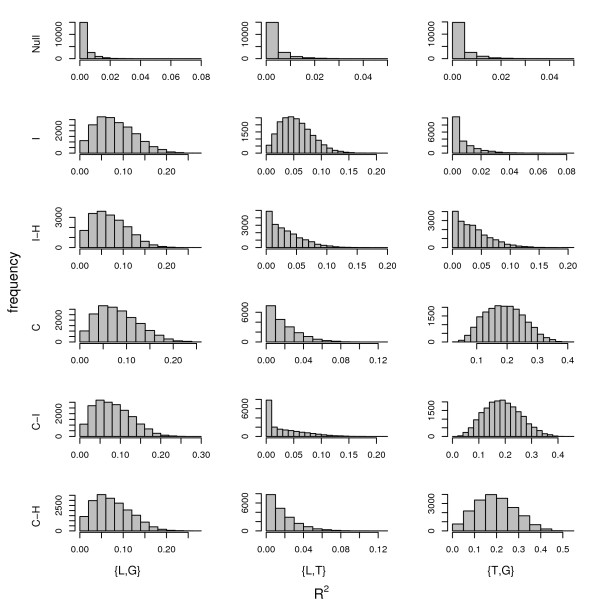
**Marginal effect sizes (sample R^2 ^values) for all six causality scenarios**. Causal models are, causal (C), reactive (R), independent (I), hidden variable affecting both traits (H), and no associations between genotypes and traits (Null). R^2 ^values are shown for all replicate datasets within each causal scenario.

One thousand replicate crosses were simulated under each of six causality scenarios using equations 1–3 (Figure 4). While the form of each linear model within each causal scenario was identical for all chromosomes and all replicate crosses, parameter values were unique to each chromosome/cross combination. Parameter values were randomly selected from a uniform distribution and randomly assigned a positive or negative sign (see Figure 5 for coefficients of determination for all six causality scenarios). Percent variance in the gene trait explained by the locus was generally less then 25%, whereas percent variance in the clinical trait explained by the locus was less than 15%. Genotype effects were simulated according to an additive model.

#### Causal scenarios

A) Null. Trait data were sampled from a normal distribution and were completely independent of genotype data.

B) Independent. Data were simulated according to equations 2 and 3, with {*β*_3_, *β*_6_} fixed at zero.

C) Independent/hidden variable. This scenario is like B but with the inclusion of an additional normal covariate term to simulate a hidden factor.

D) Causal. Data were simulated according to equations 2 and 3, with {*β*_3_, *β*_7_, *β*_8_} fixed at zero. Thus, the association between L and T is entirely mediated by G.

E) Causal/independent. This scenario is like D but only *β*_3 _is fixed at zero. Parameters {*β*_7_, *β *_8_} were randomly assigned small positive or negative non-zero values.

F) Causal/hidden. This scenario is like D but with the inclusion of an additional normal covariate term to simulate a hidden variable.

**Figure 6:**
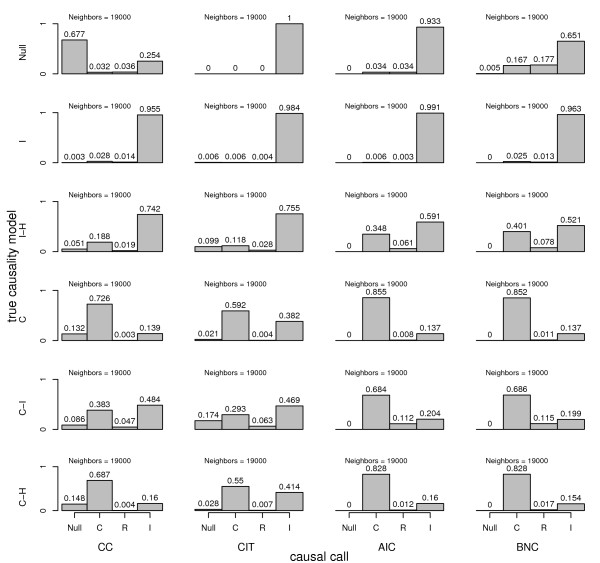
**Type I error and power comparison between causality methods derived from computer simulated F2 mouse crosses**. For each autosome of each replicate cross of N = 1000 total crosses, a clinical trait and potential mediating trait were simulated under a variety of true causal scenarios. For each scenario, a wide range of positive and negative effect sizes were randomly selected for each chromosome of each cross. 'Neighbors' denote chromosome-specific QTL peak pairs. Causal models are, causal (C), reactive (R), independent (I), hidden variable affecting both traits (H), and no associations between genotypes and traits (Null).

Simulation results (Figure 6) show type I error under the null scenario to be problematic for the BNC method, with about 35 percent false causal and reactive calls. This poor performance is probably due to the fact that equal priors are used for all three causal models. Setting a higher prior for the independence model may solve this problem, but a consequence would undoubtedly be a reduction in power. Another difficultly with this alternative approach would be deciding exactly what priors to use. Another alternative approach would be to bootstrap the analysis and choose a consistency threshold to accept the causal call. Similarly, this alternative approach would decrease the false positives but also decrease the power, and there would be the difficulty of choosing an appropriate threshold value. A threshold chosen for one set of conditions may not be optimal for another set of conditions. In practice, the analyst can avoid this scenario by requiring evidence of statistically significant eQTL and cQTL before proceeding with the model selection step, thereby insuring the independent scenario in cases were gene expression (or other potential mediator) is not linked to the clinical trait.

The CC and AIC methods performed similarly under the null scenario, both yielding approximately 7 percent of false causal and reactive calls. While slightly elevated, this is closer to the target 0.05 type I error rate. The CIT clearly demonstrated the lowest type I error under the null scenario, yielding zero false positives. This result highlights one of the positive attributes of the CIT, the formal integration of diverse criteria into the final causal inference. Thus, the CIT is robust to the choice of prior filters applied to the data. Unlike more standard statistical tests that are designed to function under a single null distribution, the CIT must be robust to an array of null conditions that include the null, independent, and independent-hidden, scenarios. It is understandable then, that depending on conditions the CIT may over or undershoot the target type I error.

Under the simple independence model, type I error for the CIT and the other methods is low, and all four methods are conservative under these conditions. The CIT and AIC are significantly more conservative under this scenario than the CC and BNC methods, with about 3 percent fewer false positives. All methods have more false causal calls than false reactive calls, an indication that a potential mediator is more likely to be called 'causal' if the eQTL is more significant than the cQTL.

The independent-hidden scenario revealed clearly challenging conditions, with all four methods surpassing the .05 type I error target by a significant amount. The CIT had the lowest type I error, 15 percent, which was less than half that of the AIC, 41 percent, and the BNC, 48 percent. The CC fell between these two groups, 21 percent. As discussed above, type I error for all methods could be improved by applying various filtering criteria, however, it is not trivial to determine the global optimal thresholds.

Qualitatively similar differences in power between methods were observed for the causal, causal-independent, and causal-hidden scenarios. The CC had more power than the CIT, and the AIC and BNC had more power than the CC. The performances of the AIC and BNC were almost identical under these conditions. The largest spread in power was observed for the causal-independent scenario, which resulted in approximately 69 percent power achieved for AIC and BNC methods compared with 29 percent for the CIT and 38 percent for the CC. This spread reflects the challenge for the CC and CIT methods of satisfying the conditional independence criterion.

**Figure 7:**
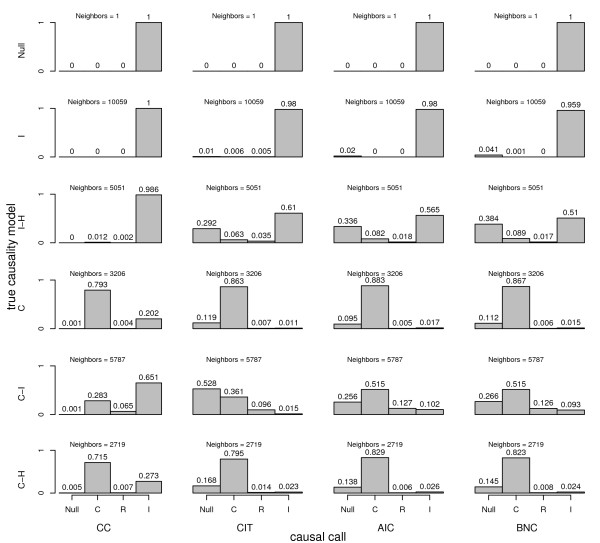
**Type I error and power comparison between causality methods derived from computer simulated F2 mouse crosses**. For each autosome of each replicate cross of N = 1000 total crosses, a clinical trait and potential mediating trait were simulated under a variety of true causal scenarios. For each scenario, a wide range of positive and negative effect sizes were randomly selected for each chromosome of each cross. 'Neighbors' denote chromosome-specific QTL peak pairs. Causal models are, causal (C), reactive (R), independent (I), hidden variable affecting both traits (H), and no associations between genotypes and traits (Null). Filtering criteria were applied such that only neighbors where both QTL peaks achieved a p-value of .001 or smaller were tested. For the AIC and BNC methods, a bootstrap consistency of .7 was required to accept the causal call. Note that 'power' is estimated ignoring those gene-trait pairs that did not both meet the p-value significance threshold.

**Figure 8:**
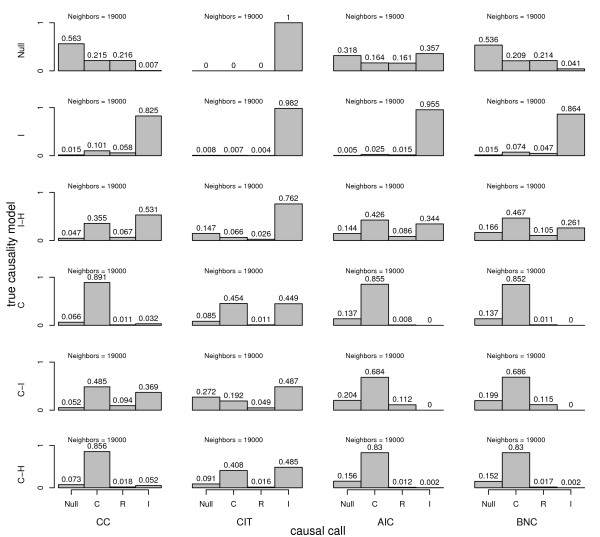
**Type I error and power comparison between causality methods derived from computer simulated F2 mouse crosses**. For each autosome of each replicate cross of N = 1000 total crosses, a clinical trait and potential mediating trait were simulated under a variety of true causal scenarios. For each scenario, a wide range of positive and negative effect sizes were randomly selected for each chromosome of each cross. 'Neighbors' denote chromosome-specific QTL peak pairs. Causal models are, causal (C), reactive (R), independent (I), hidden variable affecting both traits (H), and no associations between genotypes and traits (Null). Unlike all other results reported here, the causal model was tested using the gene QTL peak marker and the reactive model was tested using the clinical trait QTL peak marker.

In terms of sensitivity and specificity, we observed that the methods with higher sensitivity had lower specificity and vice versa. There is a trade-off between sensitivity and specificity, and it is often possible to adjust the mix using tuning parameters or filtering devices. If type I error is approximately adjusted by filtering out QTL with p-values greater than 0.001 and requiring a bootstrap consistency of greater than .7 for the AIC and BNC methods, then power is similar for the CIT, AIC, and BNC methods for the causal and causal-hidden scenarios. Under the causal-independent scenario, the AIC and BNC method are more powerful than the CC and CIT methods (Figure 7). Finally, if the causal model is tested at the gene peak and the reactive model tested at the clinical trait peak, the type I error problem observed in Figure 1 is exacerbated for all methods except the CIT, which actually has a smaller type I error for the independent-hidden scenario (Figure 8).

Thus, by creating a model selection method from the CIT, we were able to compare it to existing methods and demonstrate the conservative character of the test under most conditions without the need for heuristic filtering procedures. The intent here was not to supplant existing causal inference model selection methods with the CIT but rather to provide a complimentary statistic to quantify uncertainty in the causal call.

### Application – predictive power of the causal network

**Figure 9:**
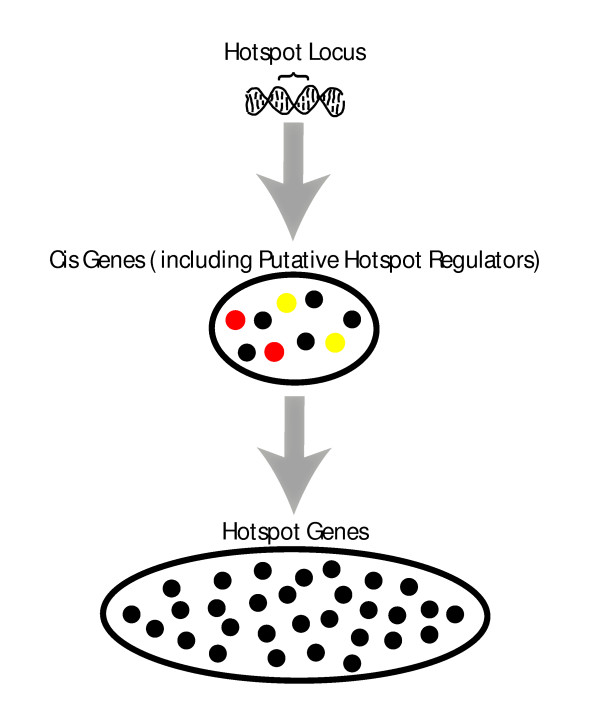
**Schematic of an eQTL hotspot, a locus identified to affect transcript abundances for many genes**. Directly affected are genes in cis, some of which, the 'cis regulators', propagate the 'perturbation' to other genes. Among cis regulated genes are the putative cis regulators identified by BN.full (yellow) as well as targeted in vivo experimentation (red).

We used the CIT to construct a causal transcriptional regulatory network for previously published genotypic and expression data from yeast by defining a directed edge between two genes if one of the genes tested significantly as a causal mediator for the other but not vice versa. One objective way to assess the relative predictive power of several networks is to use the networks to infer the causal regulators for previously identified eQTL hot spots [10]. Like transgenics, gene knockouts and other artificial perturbations, eQTLs represent perturbations that affect gene expression traits. In some cases, a given QTL may have pleiotropic effects on a number of expression traits, leading to eQTL clusters that colocalize to a common genetic locus (known as an eQTL hot spot). To identify causal regulators for a given hot spot, we selected genes with cis eQTL in the corresponding eQTL hot spot region (Figure 9). For this set of candidate regulators, we defined the signature for each as the set of genes in the subnetwork that could be reached by the candidate regulator following directed links downstream through the network. The signature for each candidate regulator was then intersected with the set of genes linked to the corresponding hot spot region. If the overlap was significant, we declared the candidate regulator to be a regulator of the hot spot and the associated subnetwork.

**Figure 10:**
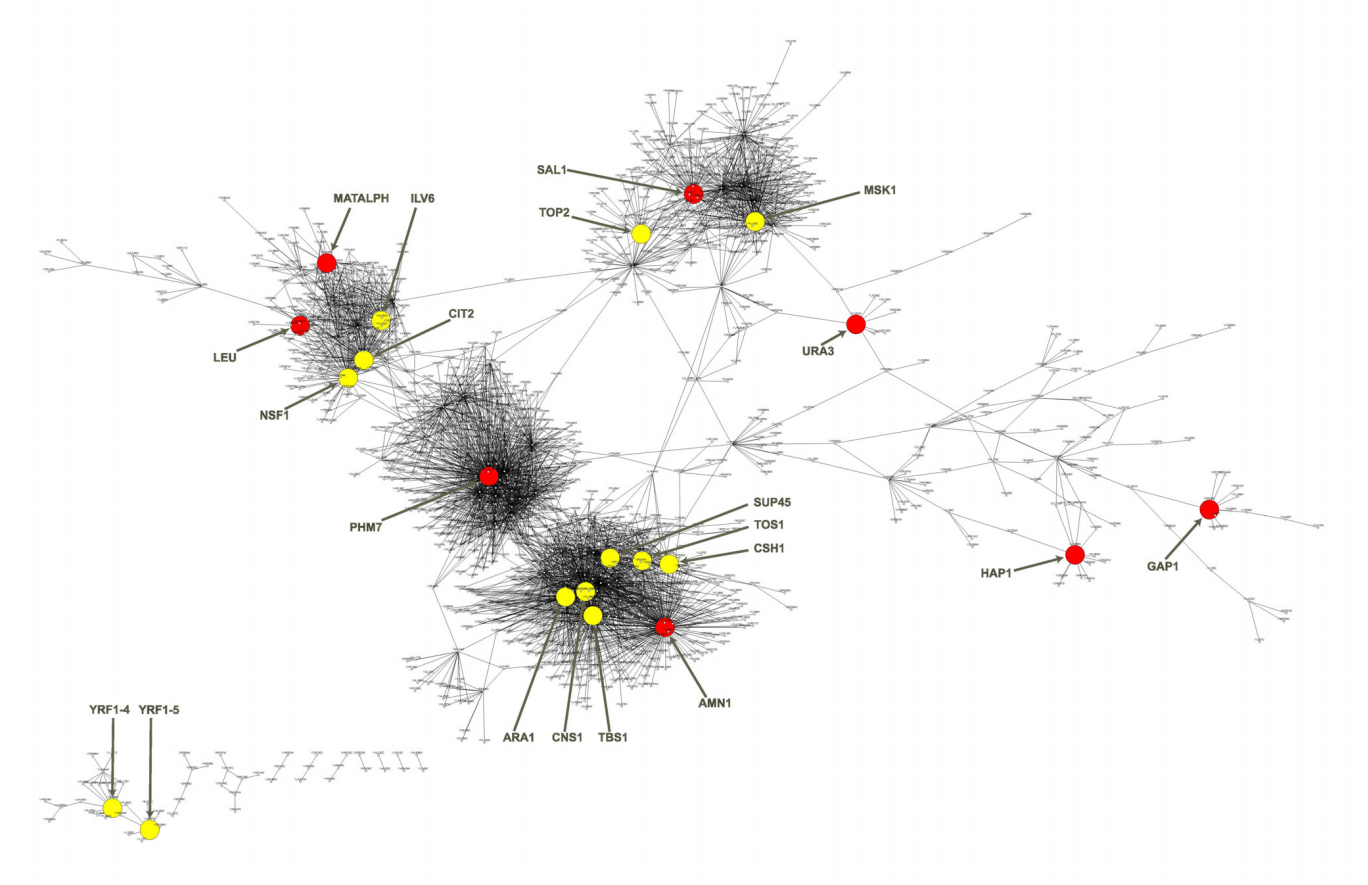
**CIT reconstructed causal transcriptional regulatory network**. Yellow circles indicate putative hotspot regulators from Table 1, and red circles indicate those that have been experimentally validated.

The yeast cross dataset (see Brem et al. for full methods [19]) includes genotypic and expression data from 112 segregants obtained from a yeast cross between the BY and RM strains of *S. cerevisiae *(referred to here as the BXR cross), and previous work has identified 9 putative regulators for 8 of the 13 hot spots based on known gene biological functions and cis eQTLs under these hot spots [19]. We identified all of the gene expression traits linked to each of the 13 hot spot regions and then searched each of these gene sets for enrichment in subnetwork structures in the CIT derived causal network that includes 1346 genes and 3965 directed links (Figure 10).

We compared the CIT to the CC, and Bayesian networks [10]. To insure a fair comparison of methods, we included a comparison to the Bayesian network, BN, based solely on gene expression and genotypic data without integrating transcription factor binding site (TFBS) and protein-protein interaction data. The idea here was to control input information so resulting differences in performance due to methodology were not confused with differences due to input information.

We assessed the predictive power of causal networks to identify the known causal regulators for the 13 eQTL hot spots. Table 1 presents p-values for regulator signature and hotspot gene set overlaps in a quantitative comparison of the causal networks. We considered the key regulators identified by BN.full, the Bayesian network with integrated transcription factor binding site and protein-protein interaction data, and determined whether their downstream genes were enriched in genes linked to QTL hot spots. In the table, each entry represents the p-value for an enrichment test of genes linked to a hotspot (column 1) in the downstream genes of a putative regulator (columns 2–4) in a causal network. All p-values have been adjusted against randomization [10]. For the 19 putative regulators (excluding YRF-4 and YRF-5, which are regulators of an artifact hotspot [10]), the CIT and the CC performed equally well (p = 0) for 10 regulators, the CIT resulted in more significant p-values for 3 regulators, but less significant for 5; the CIT and the BN.full performed equally well for 7 and the CIT led to more significant p-values for 6 but less significant for 4. The CIT missed one causal regulator, URA3, which was identified by both the CC and both of the Bayeisan networks. On the other hand, the CIT clearly out-performed BN which identified 9 regulators among the 19.

**Table 1:** P-values for overlap of putative transcriptional regulators of eQTL hotspots identified in yeast

			Bayesian	Bayesian		
Hot spot	Gene Symbol	Gene Chrom.	Full	Network	CIT	CC
Chr 2 560000	SUP45	2	0		0	2.04E-99
Chr 2 560000	ARA1	2	0		0	0
Chr 2 560000	TBS1	2	0		0	0
Chr 2 560000	CNS1	2	0		0	0
Chr 2 560000	AMN1	2	7.74E-73	1.52E-99	0	0
Chr 2 560000	CSH1	2	0		7.16E-21	7.46E-106
Chr 2 560000	TOS1	2	0		8.12E-287	0
Chr 3 1e+05	ILV6	3	1.57E-182		0	6.01E-147
Chr 3 1e+05	NFS1	3	2.04E-40		0	0
Chr 3 1e+05	LEU2	3	0	0	0	0
Chr 3 1e+05	CIT2	3	7.67E-43		0	0
Chr 3 1e+05	MATALPHA1	3	4.66E-63	9.18E-91	0	0
Chr 5 130000	URA3	5	2.04E-145	1.01E-157		3.74E-49
Chr 8 130000	GPA1	8	0	9.00E-307	1.69E-11	6.41E-07
Chr 12 680000	HAP1	12	0	0	3.63E-45	4.43E-83
Chr 12 107000	YRF1-4	12	0		0	
Chr 12 107000	YRF1-5	12	0	0		
Chr 14 503000	MSK1	14	1.39E-08	9.65E-09	2.96E-108	1.46E-141
Chr 14 503000	SAL1	14	0		0	0
Chr 14 503000	TOP2	14	1.51E-30	2.74E-73	5.56E-169	1.73E-174
Chr 15 180000	PHM7	15	0	4.48E-289	0	0

## Discussion

The CIT is the first method that we know of for computing a p-value for a potential causal mediator. By quantifying the evidence of causal mediation status it provides a tool for 1) ranking a large number of potential mediators, 2) communicating the strength of evidence to other researchers as well as the greater scientific community, and 3) making go-no-go decisions regarding future research. A user-friendly R script, implementing the CIT as a function (see Additional file 1, is provided to facilitate the broad use of this approach. We have shown that, in comparison to the other methods considered, the CIT is much more robust to underlying relationships between variables, including the significance of the gene and trait QTL as well as the choice of the test marker

We have shown that by using the CIT as a model selection method, the results are conservative yet comparable to other diverse model selection approaches when the type I error rate is controlled. We have also shown that type I error for the CIT is well controlled under the independence model and lower than other methods when a hidden variable affects both the gene and the trait. The introduction of hidden variables is challenging for most statistical techniques, however, in the case of transcriptional regulatory networks, this issue is particularly important in view of known and possibly unmeasured molecular species that can affect transcript abundance. We have shown that the CIT is robust to a variety of conditions without the need for additional heuristic filters.

When eQTL and cQTL peaks are close, but not occurring at precisely the same marker, the investigator is faced with the problem of choosing a test marker between those peaks, inclusive. We have decided that evidence and logic favors the trait peak marker. Schadt et al. [7] also tested candidate mediators at the trait peak marker, where the trait was omental fat pad mass in mouse. Chen et al. [1] in reconstructing transcriptional regulatory networks, used the marker at the mediator QTL peak. Type I error for the CC, AIC, and BNC was inflated when the potential mediator peak marker was used as the test marker as compared to the trait peak marker. However, the CIT actually exhibited lower type I error (and lower power) under these conditions, possibly due to excessively weak locus-trait associations at the mediator peak. This result is a nice demonstration of the robustness of the CIT. To test whether the potential mediator explains the full observed locus-trait association, it necessary to use the marker with the strongest observed effect, and this is the trait peak marker.

It has been demonstrated that additional information such as TFBS and protein-protein interaction data can increase the predictive power of the network [10], however, we consider the question of how best to integrate this information with causal inference methods an open research question. Bayesian methods are naturally suited to accounting for diverse sources of information through priors, as demonstrated here with the BN.full. We showed that the CIT compared favorably to BN.full and out-performed the BN approach. However, not only could one use purely Bayesian network methodology to integrate this type of additional information, one could also include CIT or CC results in a Bayesian analysis as priors (work we are planning for a future paper). Another potential strategy would be to use TFBS predictors derived from genomic data such as eQTL mapping results [20], as a filtering device for selecting candidate gene pairs for subsequent CIT testing.

There are several possible reasons for the strong performance of the CC and CIT relative to the BN. First, the BN is optimized based on a global objective function yet all methods are evaluated on a local level. It is still possible that the BN out-performed the other methods on a global scale that was not evaluated (due to the difficulty of such an evaluation). Secondly, the BN approach requires a limit to the number of parents each node can have (five), as well as the requirement of no feedback loops, which are not required for the other methods. Third, it is possible that an adjustment of priors could improve the performance of the BN approach.

The main caveat to all causal inference approaches considered here, is that there are limitations in our ability to make causal inferences for any variable that is not experimentally perturbed. For instance, a potential mediator that tests positive may be acting as a surrogate for a tightly linked unmeasured variable that is truly causal. However, even in such a case the evidence of causality may help direct the research to the true unmeasured factor.

We emphasized a semi-parametric approach here, which is useful for high throughput data where computational resources are often limiting, but the approach does require the reliance on some distributional assumptions. However, each component p-value of the CIT could be computed using permutation tests, which would yield a completely non-parametric approach. Alternatively, the probit transformation could be used for the gene and the trait to approximate normality, which is a valid approach under large-sample conditions [1]. Also, we assumed a linear relationship between the two quantitative traits, which may not always yield the best fit but allows the approach to be general, an advantage when the true relationship is unknown.

It's important to note that this is a flexible framework that can be generalized to handle complex eQTL methods that require the use of covariates and complex data structures [21-23] as well as multilocus effects. For instance, rather than F statistics, an analogous approach could be taken with chi square statistics from likelihood ratio tests, which would extend the method to logistic regression and generalized linear models with other link functions.

## Conclusion

As microarray gene expression profiling and other high-throughput technologies for measuring intracellular molecular traits become more commonly used, there is increasing demand for statistical tools that distinguish between competing causal models such as pleiotropic (independence) and reactive transcriptional control. The CIT is unique in that it provides a highly interpretable quantitative measure of uncertainty in the form of a formal p-value that can be computed for a trio of variables when association between two implies causation and the third is a potential mediator. The CIT can be conducted as building block for a network reconstruction problem or as an isolated test apart from a larger network.

## Authors' contributions

JM designed and implemented the CIT, performed the computer simulations, and wrote the manuscript. BZ performed all statistical analyses for the Application section, wrote the Application section, and contributed to the concept and editing of the manuscript. EES and JZ contributed to the concept, writing, and editing of the manuscript. All authors read and approved the final manuscript.

## Supplementary Material

Additional file 1 **CIT R script**. This file includes a script to implement the CIT as a function in R. Click here for file
