# Supplementary material for: Disentangling molecular relationships with a causal inference test
Source: BMC Genet. 2009 May 27;10:23. doi: 10.1186/1471-2156-10-23 (PMC3224661; doi:10.1186/1471-2156-10-23)
Supplement: Additional file 1 — CIT R script. This file includes a script to implement the CIT as a function in R. [file 1471-2156-10-23-S1.doc]

**Disentangling molecular relationships with a causal inference test**

Joshua Millstein, Bin Zhang, Jun Zhu, Eric E. Schadt

CIT algorithm (R script)

########################################################################

#Causal Inference Test -- Intersection-Union Test;

#Purpose: hypothesis test for potential causal mediator

#Programmer: Joshua Millstein

#

#Example call:

#L = rbinom(100,2,.5)

#G = + L + rnorm(100)

#T = + G + rnorm(100)

#CausalityTestJM(L,G,T)

#

#Output:

#pvalc = p-value for causal model L -> G -> T

#pvalr = p-value for reactive call L -> T -> G

#ccall = causal call 0:no call, 1:causal, 2:reactive, 3:independent(or other)

CausalityTestJM = function(LL,GG,TT){

no.bootstrap = 50

# remove missing values

sel = (!is.na(LL)) & (!is.na(GG)) & (!is.na(TT))

dat_f = as.data.frame(cbind(LL,GG,TT),stringsAsFactors=FALSE)

dat_f = dat_f[sel,]

names(dat_f) = c("L","G","T")

Lf = as.factor(dat_f$L)

dat_f$L = as.integer(Lf) - 1

llevels = as.integer(levels(as.factor(dat_f$L)))

dfL = length(llevels) - 1

pvec = rep(NA,4)

if(dfL == 2){

dat_f$L1 = ifelse(dat_f$L == 1,1,0)

dat_f$L2 = ifelse(dat_f$L == 2,1,0)

fit0 = lm(T ~ 1,data=dat_f)

fit1 = lm(T ~ L1 + L2,data=dat_f)

fit2 = lm(G ~ T,data=dat_f)

fit3 = lm(T ~ G,data=dat_f)

fit4 = lm(G ~ T + L1 + L2,data=dat_f)

fit5 = lm(T ~ G + L1 + L2,data=dat_f)

pvec[1] = anova(fit0,fit1)$"Pr(>F)"[2]

pvec[2] = anova(fit2,fit4)$"Pr(>F)"[2]

pvec[3] = anova(fit1,fit5)$"Pr(>F)"[2]

f_ = anova(fit3,fit5)$F[2]

fit1G = lm(G ~ L1 + L2,data=dat_f)

alg = summary(fit1G)$coefficients["(Intercept)",1]

blg1 = summary(fit1G)$coefficients["L1",1]

blg2 = summary(fit1G)$coefficients["L2",1]

alt = summary(fit1)$coefficients["(Intercept)",1]

blt1 = summary(fit1)$coefficients["L1",1]

blt2 = summary(fit1)$coefficients["L2",1]

dat_f$eG = resid(fit1G)

dat_f$eT = resid(fit1)

ss = dim(dat_f)[1]

fvecr = rep(NA,no.bootstrap)

fvecr_r = rep(NA,no.bootstrap)

for(i in 1:no.bootstrap){

nni <- trunc(1 + ss*runif(ss, 0, 1)) ;

dat_f$G_ = alg + blg1*dat_f$L1 + blg2*dat_f$L2 + dat_f$eG[nni]

fit_0 = lm(T ~ G_,data=dat_f)

fit_1 = lm(T ~ G_ + L1 + L2,data=dat_f)

fvecr[i] = anova(fit_0,fit_1)$F[2]

dat_f$T_ = alt + blt1*dat_f$L1 + blt2*dat_f$L2 + dat_f$eT[nni]

fit_0 = lm(G ~ T_,data=dat_f)

fit_1 = lm(G ~ T_ + L1 + L2,data=dat_f)

fvecr_r[i] = anova(fit_0,fit_1)$F[2]

}

}#End dfL == 2

if(dfL == 1){

dat_f$L1 = ifelse(dat_f$L == 1,1,0)

fit0 = lm(T ~ 1,data=dat_f)

fit1 = lm(T ~ L1,data=dat_f)

fit2 = lm(G ~ T,data=dat_f)

fit3 = lm(T ~ G,data=dat_f)

fit4 = lm(G ~ T + L1,data=dat_f)

fit5 = lm(T ~ G + L1,data=dat_f)

pvec[1] = anova(fit0,fit1)$"Pr(>F)"[2]

pvec[2] = anova(fit2,fit4)$"Pr(>F)"[2]

pvec[3] = anova(fit1,fit5)$"Pr(>F)"[2]

f_ = anova(fit3,fit5)$F[2]

fit1G = lm(G ~ L1,data=dat_f)

alt = summary(fit1)$coefficients["(Intercept)",1]

blt1 = summary(fit1)$coefficients["L1",1]

alg = summary(fit1G)$coefficients["(Intercept)",1]

blg1 = summary(fit1G)$coefficients["L1",1]

dat_f$eG = resid(fit1G)

dat_f$eT = resid(fit1)

ss = dim(dat_f)[1]

fvecr = rep(NA,no.bootstrap)

fvecr_r = rep(NA,no.bootstrap)

for(i in 1:no.bootstrap){

nni <- trunc(1 + ss*runif(ss, 0, 1)) ;

dat_f$G_ = alg + blg1*dat_f$L1 + dat_f$eG[nni]

fit_0 = lm(T ~ G_,data=dat_f)

fit_1 = lm(T ~ G_ + L1,data=dat_f)

fvecr[i] = anova(fit_0,fit_1)$F[2]

dat_f$T_ = alt + blt1*dat_f$L1 + dat_f$eT[nni]

fit_0 = lm(G ~ T_,data=dat_f)

fit_1 = lm(G ~ T_ + L1,data=dat_f)

fvecr_r[i] = anova(fit_0,fit_1)$F[2]

}

} #End dfL == 1

#####F Method

fvecr = fvecr[!is.na(fvecr)]

df1 = anova(fit3,fit5)$Df[2]

df2 = anova(fit3,fit5)$Res.Df[2]

fncp = mean(fvecr,na.rm=TRUE)*(df1/df2)*(df2-df1)-df1

if(fncp < 0) fncp = 0

######### Transform F to normal

npvals = pf(fvecr,df1,df2,ncp=fncp,lower.tail=TRUE)

nfvecr = qnorm(npvals)

npf = pf(f_,df1,df2,ncp=fncp,lower.tail=TRUE) #Transform observed F

zf = qnorm(npf)

pvec[4] = pnorm(zf,mean=0,sd=sd(nfvecr))

pvalc = max(pvec) ###Causal p-value

#### Reactive p-value

fit0G = lm(G ~ 1,data=dat_f)

pvec1 = rep(NA,4)

pvec1[1] = anova(fit0G,fit1G)$"Pr(>F)"[2]

pvec1[2] = anova(fit3,fit5)$"Pr(>F)"[2]

pvec1[3] = anova(fit1G,fit4)$"Pr(>F)"[2]

f_ = anova(fit2,fit4)$F[2]

#####F Method

fvecr_r = fvecr_r[!is.na(fvecr_r)]

df1 = anova(fit3,fit5)$Df[2]

df2 = anova(fit3,fit5)$Res.Df[2]

fncp = mean(fvecr_r,na.rm=TRUE)*(df1/df2)*(df2-df1)-df1

if(fncp < 0) fncp = 0

######### Transform F to normal

npvals = pf(fvecr_r,df1,df2,ncp=fncp,lower.tail=TRUE)

nfvecr = qnorm(npvals)

npf = pf(f_,df1,df2,ncp=fncp,lower.tail=TRUE) #Transform observed F

zf = qnorm(npf)

pvec1[4] = pnorm(zf,mean=0,sd=sd(nfvecr))

pvalr = max(pvec1) ###Reactive p-value

ccall = NA

ccall = ifelse((pvalc < .05) & (pvalr > .05),1,ccall)

ccall = ifelse((pvalc > .05) & (pvalr < .05),2,ccall)

ccall = ifelse((pvalc > .05) & (pvalr > .05),3,ccall)

ccall = ifelse((pvalc < .05) & (pvalr < .05),0,ccall)

return(c(pvalc,pvalr,ccall))

}
